# Editorial: Case reports in pulmonary medicine 2024

**DOI:** 10.3389/fmed.2025.1680815

**Published:** 2025-08-26

**Authors:** Karolina H. Czarnecka-Chrebelska, Uday Kishore

**Affiliations:** ^1^Department of Biomedicine and Genetics, Medical University of Lodz, Lodz, Poland; ^2^Department of Veterinary Medicine (CAVM) and Zayed Centre for Health Sciences, United Arab Emirates University, Al Ain, United Arab Emirates

**Keywords:** severe lung infections, pulmonary infection, Pneumonia—clinical features and management, next-generation sequencing—NGS, *Chlamydia psittaci* (*C. psittaci*), *Penicillium digitatum*, *Aeromonas dhakensis* AS3

Recent clinical reports highlight a growing trend of unusual pulmonary infections affecting both immunocompetent and immunocompromised patients. Atypical pathogens such as *Chlamydia psittaci, Penicillium digitatum, Nocardia*, and *Aeromonas dhakensis* are increasingly recognized as causes of severe respiratory illnesses, often resulting in life-threatening complications. Identifying the infectious agent and reaching a final diagnosis are often delayed because initial symptoms and clinical signs are non-specific, resembling common respiratory infections. This similarity makes it difficult for healthcare providers to determine the exact cause of the illness promptly. Case studies show that traditional diagnostic tools often fall short, leading to delays and sometimes inappropriate treatments. The use of metagenomic next-generation sequencing (mNGS) has proven invaluable in detecting rare pathogens and guiding personalized therapies. However, the high cost of NGS and the need for rapid initiation of clinical antimicrobial treatment still contribute to delays in diagnosis (1).

Several reports highlight the often-overlooked burden of *Chlamydia psittaci* pneumonia. In two case studies, patients showed classic symptoms like fever, cough, dyspnea, and chest tightness—a non-specific clinical presentation that closely resembled common community-acquired pneumonia (Zhang et al.; Yan et al.). In one case, deep vein thrombosis complicated the course of the infection, and the patient did not respond to broad-spectrum antibiotics (Zhang et al.). In another case, despite thorough laboratory and radiological testing, the cause of pneumonia remained unknown (Yan et al.). In both cases, testing bronchoalveolar lavage fluid (BALF) samples with targeted next-generation sequencing (tNGS) enabled a quick and accurate diagnosis (Zhang et al.; Yan et al.). Similarly, mNGS played a key role in promptly confirming severe Legionnaires' disease, not identified with conventional testing (Fang et al.). The case describes two instances of *Legionellosis* with different outcomes. For the elderly immunocompromised patient, the delayed diagnosis led to the continued use of broad-spectrum antibiotics, allowing the infection to worsen unchecked. In contrast, the prompt use of mNGS in the case of the middle-aged patient enabled an accurate diagnosis and eventual recovery (Fang et al.).

Fungal and parasitic infections also merit attention in this regard. The first reported case in China of invasive *Penicillium digitatum* lung infection demonstrates how environmental fungi can cross into clinical pathology (Shi et al.). The patient's symptoms and imaging (chronic cough, consolidation on CT) resembled bacterial pneumonia, and standard bacterial and fungal cultures failed to detect the pathogen. *P. digitatum* was identified only after mNGS on BALF and confirmatory targeted fungal culture, enabling tailored treatment that controlled the infection (Shi et al.).

*Nocardia—*and anthropozoonotic bacteria species have been increasingly reported as a cause of rapidly advancing pulmonary disease. Conventional diagnostic methods typically require long-lasting cultures (2–32 days) and can have low sensitivity, as fast-growing bacteria may overshadow/outnumber *Nocardia*, leading to missed detections despite strong clinical suspicion (2). Recent cases reveal that delayed *Nocardia* detection may cause life-threatening symptoms: a woman with gingival pain and pharyngeal discomfort, treated with oral metronidazole, quickly developed breathing difficulties. Her imaging resembled tuberculosis, but routine sputum and blood cultures were negative. Due to her deteriorating health, fiberoptic bronchoscopy was performed, revealing no tumor or severe inflammation. Yet, the lavage smear after a week of culture enabled the detection of *Nocardia*, allowing for targeted therapy and rapid disease control (Chen et al.). On the other hand, using NGS can provide results in about 48 h, with *Nocardia* species often ranking among the top two organisms detected (2). In the case of an immunosuppressed patient with Myasthenia gravis, 16S rRNA gene sequencing helped distinguish pulmonary nocardiosis from more common infections in an immunocompromised host, and ultimately, *Nocardia cyriacigeorgica* infection was confirmed (Zuo et al.).

In the case of a 19-year-old patient who presented with a simple sore throat but then deteriorated into septic shock with multiple serous effusions, repeated sputum cultures were negative. The mNGS analysis of blood and pericardial fluid led to the identification of the pathogen—anaerobe *Prevotella oris* (a member of the oral cavity microbiome, regarded as commensals in the oral cavity) that caused systemic pleural infection (Zhang et al.). Another case of pneumonia, which rapidly worsened to septic shock and severe pulmonary hypertension, was caused by the anaerobic pathogen *Aeromonas dhakensis*. Due to the severe acute respiratory distress syndrome, the mNGS of BALF and blood was performed promptly that subsequently led to the initiation of *A. dhakensis-*targeted treatment (Sha et al.). Rare human pathogen—zoonotic *Chlamydia abortus* can mimic community-acquired pneumonia, as seen in the 74-year-old female, who worsened rapidly. Standard bacterial, fungal, and viral tests were repeatedly negative, and a definitive diagnosis required mNGS on BALF, ultimately guiding effective targeted therapy (Yang and Shu). Similarly, a patient initially suffering from allergic and pulmonary infections, treated with allergic medications and broad-spectrum antibiotics for over a year, eventually developed symptoms that resembled tuberculosis both clinically and radiographically. After specialized culture and molecular testing, the diagnosis revealed the rare non-tuberculous mycobacteria—*Mycobacterium riyadhense* (Sawan et al.).

Pulmonary infection symptoms can sometimes result from severe parasitic invasion, as seen in a 75-year-old immunocompromised patient presenting with non-specific respiratory symptoms like cough and shortness of breath (Fang et al.). Initially, the diagnosis indicated a common bacterial or fungal pulmonary infection; however, symptoms persisted and worsened despite treatment. mNGS analysis of BALF samples detected *E. faecium, C. albicans, C. glabrata*, and unexpectedly, *Strongyloides stercoralis*—a soil-transmitted nematode common in tropical and subtropical regions (Fang et al.). Notably, uncomplicated strongyloidiasis is often asymptomatic, while severe cases can cause abdominal pain, diarrhea, vomiting, nausea, colitis, and gastrointestinal bleeding signs, though respiratory symptoms are rare (Fang et al.).

The commented cases highlight the importance of prompt pathogen identification, which enables targeted antibiotic therapy. This approach reduces the severity and duration of illness, prevents progression to multi-organ failure, and ultimately allows for complete recovery and hospital discharge. Some cases revealed novel associations, such as deep vein thrombosis secondary to psittacosis or fatal progression from oral infection. These findings stress the importance of considering rare etiologies in atypical pneumonias and support broader adoption of molecular diagnostics. In immunocompromised patients or those experiencing rapid clinical deterioration, early incorporation of genomic analysis—particularly metagenomic next-generation sequencing (mNGS)—is critical for timely and accurate pathogen identification when conventional diagnostics fail. This approach enables prompt, targeted treatment, which can significantly improve outcomes and, in many cases, be life-saving.

In the evolving field of respiratory medicine, recent case reports show an increasingly diverse range of pathogens responsible for severe lung infections. These cases—often involving rare or emerging microorganisms—highlight the diagnostic challenges of traditional methods and the clinical impact of delayed or incorrect treatment. On one hand, it is noted that the lungs are becoming increasingly susceptible to a broader range of emerging pathogens, including zoonotic bacteria and environmental fungi. On the other hand, more detailed analysis of clinical symptoms (with broader biomarker testing) and the use of advanced diagnostic tools could offer more precise and comprehensive ways to identify these threats early, leading to better clinical outcomes. Together, these cases emphasize the diagnostic and therapeutic importance of metagenomic next-generation sequencing (mNGS). By enabling rapid, unbiased pathogen detection, mNGS plays a transformative role in modern infectious disease management, particularly when traditional methods may overlook pathogens.
